# A CRISPR Activation Screen Identifies an Atypical Rho GTPase That Enhances Zika Viral Entry

**DOI:** 10.3390/v13112113

**Published:** 2021-10-20

**Authors:** Anh Phuong Luu, Zhenlan Yao, Sangeetha Ramachandran, Stephanie A. Azzopardi, Linde A. Miles, William M. Schneider, H.-Heinrich Hoffmann, Leonia Bozzacco, Gustavo Garcia, Danyang Gong, Robert Damoiseaux, Hengli Tang, Kouki Morizono, Charles M. Rudin, Ren Sun, Vaithilingaraja Arumugaswami, John T. Poirier, Margaret R. MacDonald, Charles M. Rice, Melody M. H. Li

**Affiliations:** 1 Department of Microbiology, Immunology, and Molecular Genetics, University of California, Los Angeles, CA 90095, USA; phuonganhluu94@gmail.com (A.P.L.); jadeyao89@g.ucla.edu (Z.Y.); sangiram18@g.ucla.edu (S.R.); 2 Laboratory of Virology and Infectious Disease, The Rockefeller University, New York, NY 10065, USA; azz.steph@gmail.com (S.A.A.); wschneider@rockefeller.edu (W.M.S.); hhoffmann@mail.rockefeller.edu (H.-H.H.); leonia.bozzacco@gmail.com (L.B.); macdonm@rockefeller.edu (M.R.M.); ricec@mail.rockefeller.edu (C.M.R.); 3 Human Oncology & Pathogenesis Program, Memorial Sloan Kettering Cancer Center, New York, NY 10065, USA; milesl@mskcc.org; 4 Department of Molecular and Medical Pharmacology, University of California, Los Angeles, CA 90095, USA; GustavoGarcia@mednet.ucla.edu (G.G.J.); gongdanyang@gmail.com (D.G.); RDamoiseaux@mednet.ucla.edu (R.D.); RSun@mednet.ucla.edu (R.S.); VArumugaswami@mednet.ucla.edu (V.A.); 5 California NanoSystems Institute, University of California, Los Angeles, CA 90095, USA; 6 Department of Bioengineering, Samueli School of Engineering, University of California, Los Angeles, CA 90095, USA; 7 Jonsson Comprehensive Cancer Center, University of California, Los Angeles, CA 90095, USA; 8 Department of Biological Science, Florida State University, Tallahassee, FL 32306, USA; tang@bio.fsu.edu; 9 Division of Hematology and Oncology, Department of Medicine, David Geffen School of Medicine, University of California, Los Angeles, CA 90095, USA; koukimo@g.ucla.edu; 10 AIDS Institute, David Geffen School of Medicine, University of California, Los Angeles, CA 90095, USA; 11 Druckenmiller Center for Lung Cancer Research and Department of Medicine, Thoracic Oncology Service, Memorial Sloan Kettering Cancer Center, New York, NY 10065, USA; rudinc@mskcc.org; 12 Eli and Edythe Broad Center of Regenerative Medicine and Stem Cell Research, University of California, Los Angeles, CA 90095, USA; 13 Laura and Isaac Perlmutter Cancer Center, New York University Langone Health, New York, NY 10016, USA; John.Poirier@nyulangone.org; 14 Molecular Biology Institute, University of California, Los Angeles, CA 90095, USA

**Keywords:** CRISPR activation, Zika virus, proviral factors, WWTR1, Rho GTPases, RhoV

## Abstract

Zika virus (ZIKV) is a re-emerging flavivirus that has caused large-scale epidemics. Infection during pregnancy can lead to neurologic developmental abnormalities in children. There is no approved vaccine or therapy for ZIKV. To uncover cellular pathways required for ZIKV that can be therapeutically targeted, we transcriptionally upregulated all known human coding genes with an engineered CRISPR–Cas9 activation complex in human fibroblasts deficient in interferon (IFN) signaling. We identified Ras homolog family member V (*RhoV*) and WW domain-containing transcription regulator 1 (*WWTR1*) as proviral factors, and found them to play important roles during early ZIKV infection in A549 cells. We then focused on RhoV, a Rho GTPase with atypical terminal sequences and membrane association, and validated its proviral effects on ZIKV infection and virion production in SNB-19 cells. We found that RhoV promotes infection of some flaviviruses and acts at the step of viral entry. Furthermore, RhoV proviral effects depend on the complete GTPase cycle. By depleting Rho GTPases and related proteins, we identified RhoB and Pak1 as additional proviral factors. Taken together, these results highlight the positive role of RhoV in ZIKV infection and confirm CRISPR activation as a relevant method to identify novel host–pathogen interactions.

## 1. Introduction

Flaviviruses (family *Flaviviridae*, genus *Flavivirus*) are positive-sense single-stranded RNA viruses transmitted by arthropods. Many of these viruses cause significant morbidities and mortalities in humans, including dengue virus (DENV), yellow fever virus (YFV), West Nile virus (WNV), and Zika virus (ZIKV) [1]. In the past, ZIKV, endemic to Africa, was known to only cause rash, fever, headache, arthralgia, and conjunctivitis. However, during recent outbreaks in French Polynesia (2013) and Brazil (2014–2016), infected individuals developed neurological diseases such as Guillain–Barré syndrome and meningoencephalitis [2]. Pregnant women infected with ZIKV during their first trimesters were more likely to give birth to children with severe brain abnormalities such as microcephaly, lissencephaly, or cortical calcification [3]. Despite the serious long-term consequences, there is no approved vaccine or therapy to control ZIKV infection [4].

Like all flaviviruses, ZIKV depends on host machinery to complete its lifecycle [5]. Flavivirus enters target cells by receptor-mediated endocytosis. The acidic environment in the endosome triggers viral fusion and uncoating, releasing the viral RNA (vRNA) into the cytoplasm. The vRNA is then translated into a polyprotein on the rough endoplasmic reticulum (ER), and processed by both host and viral proteases into structural (capsid (C), pre-membrane (prM), and glycoprotein envelope (E)) and non-structural (NS) proteins (NS1, NS2A, NS2B, NS3, NS4A, NS4B, and NS5). Flaviviruses extensively remodel the ER into RNA replication and virus assembly centers to produce new virions before leaving the host cell through exocytosis, thus spreading the infection to neighboring cells.

It is critical to uncover ZIKV–host interactions as they might inform novel therapeutic approaches through inhibiting host factors and pathways required for viral infection. Genome-wide screens using clustered regularly interspaced short palindromic repeats (CRISPR) combined with Cas9 nuclease and RNA interference (RNAi) to knock out or down host genes have identified a plethora of proviral factors involved in entry and endocytosis, heparin sulfate biosynthesis, ER and Golgi functions, autophagy, and interferon regulation [6,7,8,9,10]. As a complementary unbiased approach to uncover host mechanisms that interact with ZIKV, we performed a screen based on transcriptionally upregulating all known human coding genes and their splice variants using an engineered CRISPR–Cas9 activation complex named synergistic activation mediator (SAM). The SAM complex consists of a catalytically inactive Cas9 interacting with four tandem copies of herpes simplex viral protein 16 (VP64), the nuclear factor kappa-light-chain-enhancer of activated B cells (NFκB) trans-activating subunit p65, and human heat-shock factor 1 (HSF1), which recruit transcription factors and chromatin remodeling complexes to promoters targeted by a genome-wide single-guide RNA (sgRNA) library [11]. This approach provides an advantage over overexpression screens using cDNA libraries, which often overlook functional gene isoforms.

One caveat for pooled screens is that viral detection by the host cell activates type I interferon (IFN) production, leading to upregulation of a wide array of IFN-stimulated genes (ISGs) with antiviral activities [12]. ISG upregulation can mask the effects of host genes that modulate ZIKV infection and create biases during the identification of novel ZIKV–host interactions. Interestingly, another ZIKV CRISPR–Cas9 activation screen study identified mostly known antiviral ISGs [13]. Recent studies have found that ZIKV suppresses type I IFN through degradation of STAT2 [14,15], but this does not guarantee complete shutdown of the IFN response. In our screen, we used *STAT1*^−/−^ fibroblasts immortalized from patients with homozygous mutations in *STAT1* that do not respond to IFN [16] to minimize the masking effects of IFN signaling.

Here, we identified two proviral genes, *RhoV* and *WWTR1* (also known as *TAZ*), for which targeting sgRNAs were significantly depleted in ZIKV-infected cell populations. We confirmed SAM activation, and validated their proviral effects in human lung adenocarcinoma A549 cells. We then focused on validating RhoV as a novel host proviral factor for ZIKV in this study since the hippo signaling pathway, in which WWTR1 is a transcriptional coactivator, has already been shown to play a role in ZIKV replication and ZIKV-induced neuroinflammation in the *IFNAR1*^−/−^ mouse model [17]. Due to their critical role in regulating actin cytoskeleton and a plethora of cellular processes, Rho GTPases, especially RhoA, Rac1, and Cdc42, are involved in multiple steps of the viral replication cycle to overcome the plasma membrane and cortical actin barrier of the host cell. RhoV, also known as the Chp: Cdc42Hs homolog protein, is a Rho GTPase that possesses additional N- and C-terminal sequences not found in other canonical Rho GTPases and uses palmitoylation instead of prenylation to associate with the plasma membrane [18]. We confirmed that RhoV enhances ZIKV infection in SNB-19 cells, a human glioblastoma cell line relevant for ZIKV tropism, at the step of entry. RhoV proviral effects are specific to some flaviviruses, and infection of alphaviruses, positive-sense single-stranded RNA viruses that belong to the *Togaviridae* family, is not affected. We also showed that the GTPase activity of RhoV plays an important role in enhancing ZIKV infection. Furthermore, using siRNAs targeting related Rho GTPases and their effector proteins, we demonstrated that RhoB and Pak1 are potential host proviral factors for ZIKV. Taken together, we found that an atypical Rho GTPase is critical for the ZIKV lifecycle in human cells, demonstrating that the CRISPR activation platform can be efficiently used to identify novel host–virus interactions with therapeutic potential.

## 2. Materials and Methods

### 2.1. CRISPR Activation Screen and Validation of Candidate Gene Upregulation

Human CRISPR 3-plasmid activation pooled library (SAM) [11] was a gift from Feng Zhang and obtained through Addgene (cat. #1000000074). The sgRNA library consists of 70,290 guides activating every coding isoform from the RefSeq database (23,430 isoforms; 3 sgRNAs per gene). sgRNAs target sites within 200 bp upstream of the transcription start site to give the highest levels of gene upregulation [11]. The sgRNA library was amplified, and lentivirus was produced as previously described [9,19,20]. Human *STAT1*^−/−^ fibroblasts [12,16] were stably transduced with lentiviruses that encode the SAM components, dCas9-VP64 and MS2-p65-HSF1 (multiplicity of infection (MOI) = 0.3 infectious unit (IU)/cell), and selected with blasticidin (10 µg/mL) and hygromycin (450 µg/mL). To deliver the CRISPR activation library to cells, 1.2 × 10^8^ dCas9/MS2-expressing *STAT1*^−/−^ fibroblasts were transduced with lentiviruses carrying the sgRNA library (MOI = 0.3 IU/cell) to achieve ~500× sgRNA representation and selected with puromycin (0.5 µg/mL). Following 7 days of puromycin selection, cells were pooled, seeded at 4.5 × 10^6^ per T175 flask, infected with ZIKV (strain: PRVABC59 (Puerto Rico), MOI = 0.5 plaque-forming unit (PFU)/cell) or mock infected, and incubated for 14 days in triplicate samples (24 T175 flasks per condition). The infection conditions (MOI, duration of infection) were determined prior to the screen to give the maximum amount of cell killing by ZIKV infection. Cells were trypsinized to facilitate selection. When the mock-infected cells reached confluency every 3–4 days, cells were counted and re-seeded at 500× of the library (4.5 × 10^6^ per T175 flask) to maintain sgRNA diversity while the ZIKV-infected cells were passaged and all of them re-seeded.

Following 14 days of infection and cell passaging, surviving cells were collected from mock- and ZIKV-infected flasks. Genomic DNA was isolated from these cells, amplified using a two-step nested PCR approach, purified, and sequenced as previously described [9,21]. FASTQ files were processed and trimmed to retrieve sgRNA target sequences, and MAGeCK analysis [22] was carried out to identify genes with significantly enriched or depleted sgRNAs in the ZIKV-infected cells compared to the mock-infected cells as previously described [9]. Mock-infected cells control for sgRNAs that affect general cell growth and proliferation. Positively selected sgRNAs in ZIKV-infected cells likely activate antiviral genes that protect from viral infection and cell killing, whereas negatively selected sgRNAs likely activate proviral genes that facilitate viral infection and cell killing. Two genes, *RhoV* and *WWTR1,* were identified to be significantly depleted from the ZIKV-infected cells.

To confirm transcriptional activation by SAM, sgRNAs targeting the promoter regions of RhoV and WWTR1 were identified from the SAM sgRNA library. Three sgRNAs for RhoV (NM_133639; RhoV sgRNA #1: 5′-CACCGGGGTTTTCCTCCTCCTCGCC-3′ and 5-AAACGGCGAGGAGGAGGAAAACCCC-3′; RhoV sgRNA #2: 5′-CACCGTGCCTGCCTTTCCTCCTCCC-3′ and 5′-AAACGGGAGGAGGAAAGGCAGGCAC-3; RhoV sgRNA #3: 5′-CACCGTGGAGCTCCAAGAGTCACGC-3′ and 5′-AAACGCGTGACTCTTGGAGCTCCAC-3′) and three for WWTR1 (NM_001168280; WWTR1 sgRNA #1: 5′-CACCGATGACCTCCTAGTCCCTAGC-3′ and 5′-AAACGCTAGGGACTAGGAGGTCATC-3′; WWTR1 sgRNA #2: 5′-CACCGGGGTTTTCTGGAGCCGAGGT-3′ and 5′-AAACACCTCGGCTCCAGAAAACCCC-3′; WWTR1 sgRNA #3: 5′-CACCGGTAAAGTACCCATCACGCCC-3′ and 5′-AAACGGGCGTGATGGGTACTTTACC-3′) were, respectively, ligated and cloned into Lenti sgRNA(MS2)_puro backbone (Addgene, cat. #73795) linearized with BsmBI. 293T LentiX cells were transfected with lentivirus backbone expressing non-targeting (NT) sgRNA, RhoV sgRNA #1, 2, or 3, or WWTR1 sgRNA#1, 2, or 3 in DMEM 3% FBS 1X non-essential amino acid. At 6 h post-transfection, the media of these 293T LentiX cells were replaced with DMEM 3% FBS. The lentiviral supernatant was harvested 2 days later and stored at −80 °C with final concentrations of 20 µM HEPES and 4 µg/mL polybrene. A total of 100 µL of either RhoV- or WWTR1-activating sgRNA lentivirus stocks was used to transduce 400,000 dCas9/MS2-expressing *STAT1*^−/−^ fibroblasts as this lentivirus amount gave less than 30% puromycin-resistant cells, which suggests one integration per cell. dCas9/MS2-expressing *STAT1*^−/−^ fibroblasts were then treated with 0.5 µg/mL puromycin for 3 days to eliminate the untransduced cells. Lysates of NT or WWTR1 sgRNA-transduced fibroblasts were harvested so that induction of protein expression could be determined. Due to the lack of a working RhoV antibody, RNA of NT or RhoV sgRNA-transduced cells was harvested for cDNA synthesis, and qPCR was performed to quantify RhoV mRNA levels (see Section 2.9 below).

### 2.2. Cells, Plasmids, Viruses, and Infections

Human *STAT1*^−/−^ fibroblasts [12,16] and A549 (ATCC) human lung adenocarcinoma cells were cultured in Dulbecco’s Modified Eagle Medium (DMEM) supplemented with 10% FBS. SNB-19 (a gift of Dr. Hengli Tang) human glioblastoma cells were cultured in Gibco Roswell Park Memorial Institute (RPMI) 1640 Medium supplemented with 10% FBS.

Total RNA was isolated from A549 cells by RNeasy Mini Kit (Qiagen, Hilden, Germany), and reverse transcribed using ProtoScript II First Strand cDNA synthesis (NEB) and oligonucleotide primers. RhoV and WWTR1 were amplified from A549 cDNA using (5′-GTTTAAGCTTCCGCCGCGGGAGCTG-3′ and 5′-GGTAGCGGCCGCTCAAACGAAGCAGAAGAACTTCTTCC-3′) and (5′-GTTTAAGCTTAATCCGGCCTCGGCG-3′ and 5′-GGTAGCGGCCGCTTACAGCCAGGTTAGAAAGGGC-3′), respectively, and cloned into the BamHI and HindIII sites of the enhanced PiggyBac (ePiggyBac) transposon vector with a V5 or 3×FLAG at the N-terminal end [23,24]. RhoV mutants (G40V, Q89L, C234S, and G40V/C234S) were generated by QuikChange site-directed mutagenesis kit (Agilent, Santa Clara, CA, USA) (G40V primers: 5-GGTGGGCGACGTCGCCGTGGGCA-3′ and 5′-TGCCCACGGCGACGTCGCCCACC-3′; Q89L primers: 5′-GGTCAAAATCCTCCAGTCCCGCTGTGTCC-3′ and 5′-GGACACAGCGGGACTGGAGGATTTTGACC-3′; C234S primers: 5′-CTGGAAGAAGTTCTTCAGCTTCGTTTGAGCGGC-3′ and 5′-GCCGCTCAAACGAAGCTGAAGAACTTCTTCCAG-3′).

For the CRISPR SAM screen, ZIKV (PRVABC59 obtained from the CDC, Ft. Collins) was amplified in Huh-7.5 cells and its titer determined by standard plaque assay on Huh-7.5 cells. For the follow-up studies, ZIKV was generated from infectious clone (strain: PRVABC59) kindly provided by Dr. Ren Sun [25] at UCLA. The ZIKV plasmid was amplified by the 2.5 µL DNA template protocol of REPLI-g Mini kit (Qiagen) to reduce mutations with the high-fidelity DNA polymerase (this protocol was kindly provided by Dr. Erin Mcdonald at CDC). A total of 10 µg of the amplified ZIKV plasmid was linearized with ClaI and purified directly from the restriction enzyme digestion mixture using the Zymoclean Large Fragment DNA Recovery Kit (Zymo Research, Irvine, CA, USA). Viral RNA was in vitro transcribed from 200 ng linearized ZIKV plasmid using mMESSAGE mMACHINE T7 Transcription Kit (Thermo Fisher Scientific, Waltham, MA, USA). Human hepatoma Huh-7.5 cells [26] (6 × 10^6^) growing in DMEM with 10% FBS and 1× non-essential amino acids were electroporated with 4 µg of ZIKV RNA and the virus supernatant was harvested 72 h post-electroporation. The generation of other flaviviral stocks has been previously described: DENV-GFP [27] (derived from IC30P-A, a full-length infectious clone of strain 16681) and YFV 17D Venus [28] (derived from YF17D-5′C25Venus2AUbi). ZIKV and YFV titers for MOI calculations were determined in Vero and Huh-7.5 cells, respectively, and viral infections were performed as previously described [9,29].

GFP-expressing alphaviruses (Sindbis virus (SINV), o’nyong’nyong virus (ONNV), Ross River virus (RRV), and Venezuelan equine encephalitis virus (VEEV)) were generated in baby hamster kidney 21 cells (BHK-21; ATCC) as previously described [24,30]. Viral titers for MOI calculations were determined in BHK-21 cells and viral infections were performed as previously described [30].

### 2.3. RhoV and WWTR1 Knockout (KO) by CRISPR–Cas9

sgRNAs targeting exon 1 of the human *RhoV* and *WWTR1* genes were designed using the MIT Optimized CRISPR Design website (http://crispr.mit.edu/). One sgRNA for RhoV (sgRNA #1: GATGGGTACCCCGCGCGCTAC) and two sgRNAs for WWTR1 (sgRNA #1: GCGGGTGGCCGCCCGACGAGT; sgRNA #3: GGCAAGTGATCCACGTCACGC) with the least predicted off-target effects were chosen for cloning into the Cas9-encoding PX459 vector (Addgene cat. #62988). Oligos containing the sgRNA sequences (RhoV sgRNA #1: 5′-CACCGATGGGTACCCCGCGCGCTAC-3′ and 5′-AAAGTAGCGCGCGGGGTACCCATC-3′; WWTR1 sgRNA #1: 5′-CACCGCGGGTGGCCGCCCGACGAGT-3′ and 5′-AAACACTCGTCGGGCGGCCACCCGC-3′; WWTR1 sgRNA #3: 5′-CACCGGCAAGTGATCCACGTCACGC-3′ and 5′-AAACGCGTGACGTGGATCACTTGCC-3′) were ligated and cloned into PX459 linearized with BbsI. A549 cells were transiently transfected with PX459 expressing RhoV sgRNA #1 or co-transfected with PX459 expressing WWTR1 sgRNA #1 and that expressing sgRNA #3. One day after transfection, cells were selected under 1 µg/mL puromycin for 2 days to eliminate the untransfected cells. Surviving cells were diluted in DMEM with 10% FBS and seeded in a 96-well plate at 0.3 cell/well. Single cell clones were allowed to expand and treated with puromycin to select for sensitive ones, indicating that they did not integrate the sgRNA-expressing vector. A total of 27 CRISPR clones were harvested for immunoblotting to evaluate for WWTR1 protein expression, and 4 clones (9, 15, 17, and 18) with undetectable *WWTR1* protein are shown in Figure S1A. Genomic DNA was isolated from two potential *WWTR1* KO clones (15 and 17) and a 570 bp amplicon flanking the sgRNA targeting sites was amplified by PCR and sent out for MiSeq complete amplicon sequencing at Massachusetts General Hospital (MGH) Center for Computational and Integrative Biology (CCIB) DNA Core. Sequencing results confirmed that these two clones contain substitutions, insertions and/or deletions in exon 1 of *WWTR1* in all alleles (Figure S1B). Furthermore, due to the lack of a specific antibody for RhoV, we screened a larger number of CRISPR clones (40) by Sanger sequencing (Genewiz) and MiSeq complete amplicon sequencing (MGH CCIB DNA Core). Two *RhoV* KO clones (2 and 14) contain deletions in exon 1 of *RhoV* in all alleles (Figure S1B).

### 2.4. Generation and Detection of RhoV or WWTR1 Inducible Cell Lines

*RhoV* or *WWTR1* KO A549 clones were transfected with 1:1 ratio of ePiggyBac transposase, and transposon encoding for N-terminally V5-tagged human RhoV or WWTR1. The transfection was performed using Lipofectamine 3000 (Thermo Fisher Scientific) following the manufacturer’s protocol. SNB-19 cells were transfected with 1:1 ratio of ePiggyBac transposase, and transposon encoding for 3×FLAG only (empty plasmid control), or N-terminally 3×FLAG-tagged human RhoV wild type (WT) or mutants (G40V, Q89L, C234S, and G40V/C234S). The transfection was performed using X-tremeGENE 9 (Roche, Basel, Switzerland) following the manufacturer’s protocol. Cells were selected with 1 µg/mL puromycin 2 days post-transfection. Bulk resistant cells were expanded and treated with various amounts of doxycycline (Dox) (0, 0.001, 0.01, 0.1, 1, and 10 µg/mL) to determine the optimal concentration for induction with minimal off-target effects on empty plasmid control cells. A total of 1 µg/mL of Dox was used to treat RhoV and WWTR1 inducible A549 cell lines, and 0.25 µg/mL of Dox was used to treat RhoV inducible SNB-19 cell lines.

To detect RhoV and WWTR1 protein expression, cell pellets were harvested and lysed in RIPA (150 mM NaCl, 1% NP-40, 0.5% sodium deoxycholate, 0.1% SDS, and 50 mM Tris-HCl) buffer supplemented with a complete protease inhibitor cocktail (Roche). Polypeptides were resolved by SDS–polyacrylamide gel electrophoresis (SDS–PAGE) and transferred to a nitrocellulose membrane (cat. #1620112, Bio-Rad Laboratories, Hercules, CA, USA). Immunodetection was achieved with 1:500 anti-V5 (MA5-15253, Thermo Fisher Scientific), 1:20,000 anti-FLAG (clone M2, Sigma-Aldrich, St. Louis, MO, USA), or 1:2500 anti-WWTR1 (NB110-58359, Novus Biologicals, Littleton, CO, USA). The primary antibodies were detected with 1:20,000 goat anti-mouse HRP (115-035-146, Jackson ImmunoResearch, West Grove, PA, USA), or 1:20,000 goat anti-rabbit HRP (31462, Thermo Fisher Scientific). The proteins were visualized by Prometheus ProSignal Pico ECL Reagent (Genesee Scientific, San Diego, CA, USA) on Bio-Rad ChemiDoc Imager.

### 2.5. Staining and Analysis of ZIKV-Infected Cells

ZIKV-infected A549 or SNB-19 cells were harvested and fixed in 2% formaldehyde in 96-well plates. Prior to flow cytometry analysis, cells were fixed and permeabilized with 100 µL Cytofix/Cytoperm solution (BD BioSciences, Franklin Lakes, NJ, USA) at 4 °C for 15 min. Cells were washed twice with 250 µL 1× BD Perm/Wash Buffer, and then stained with Alexa Fluor 647-conjugated Flavivirus group antigen antibody (clone D1-4G2-4-15 (4G2), Novus Biologicals) at 1:125 in 25 µL BD Perm/Wash buffer at room temperature for 30 min. Cells were washed twice again, resuspended in 200 µL PBS with 2% FBS, and stored at 4 °C until flow cytometry analysis using an Attune NxT Flow Cytometer (Thermo Fisher Scientific). We used FlowJo analysis software to gate on live, singlet, mock-infected cells (Alexa Fluor 647-negative), which is then applied to all the infected cell samples to determine infection levels. Percent infected cells in Dox-treated samples was then normalized to that in the respective untreated samples to calculate the fold change in ZIKV infection.

### 2.6. Viral Entry Assay

Inducible RhoV SNB-19 cells were treated with or without Dox (0.25 µg/mL) for 24 h, and incubated with NH_4_Cl (Millipore-Sigma) at a final concentration of 35 mM in Opti-MEM in the presence of ZIKV (MOI = 0.5 PFU/cell) at various times: 10 min prior to virus addition, or 0, 30, or 60 min post virus addition. After 2 h incubation with ZIKV at 37 °C, the cells were washed with cold PBS twice to get rid of any residual virus binding to the cell surface and further cultured in RPMI complete medium with 10% FBS for 24 h. Finally, the cells were lysed in TRIzol for RNA extraction, followed by qPCR to quantify ZIKV RNA levels (see Section 2.9 below). The normalized values were used to calculate the fold change in ZIKV RNA levels relative to the average of cells treated with NH_4_Cl at 10 min prior to virus addition (C_T_ method).

### 2.7. Immunofluorescence

SNB-19 cells overexpressing 3× FLAG-tagged RhoV WT and mutants (G40V and Q89L) were seeded in a chamberslide (Nunc Lab-Tek, Thermo Fisher Scientific) and treated with 1 µg/mL Dox for 24 h to induce RhoV expression. The cells were then washed with PBS for 3 times and fixed with 4% formaldehyde for 15 min. After quenching with 30 mM glycine/PBS for 5 min, the cells were permeabilized with 0.1% Triton-100/PBS for 5 min and blocked with 3% BSA/PBS at room temperature for 2 h. Incubation with 1:200 anti-FLAG antibody (clone SIG1-25, Sigma-Aldrich) was carried out at 4 °C overnight, followed by co-staining with 1:500 Alexa Fluor 594-conjugated secondary antibody (A-11072, Thermo Fisher Scientific) and 1:1000 phalloidin-iFluor 488 (ab176753, Abcam, Cambridge, United Kingdom) at room temperature for 2 h. Antibody and reagent surplus were washed away by PBS, followed by DAPI staining for 2 min. The cells were washed 2 times with PBS and mounted on the slide. Images were taken with a ZEISS Axio Observer.Z1.

### 2.8. siRNA Knockdown of Rho GTPases and Their Effector Proteins

SNB-19 cells were reverse transfected with 25 nM of NT Silencer (Ambion, Austin, TX, USA), or siRNAs targeting *RhoU*, *Cdc42*, *RhoQ*, *RhoJ*, *Rac1*, *RhoA*, *RhoB*, *RhoC*, *Pak1* (Dharmacon; 4 siRNAs per gene) using DharmaFECT 1 Transfection Reagent (diluted 1:100 in HBSS). At 48 h post-transfection, cells were infected with ZIKV (MOI = 0.1 PFU/cell). At 24 h post-infection (h.p.i.), cells were harvested and lysed for total RNA extraction for reverse transcription and qPCR to quantify Rho GTPase knockdown and ZIKV RNA levels (see Section 2.9 below).

### 2.9. Quantitative Reverse Transcription PCR (RT-qPCR)

Total RNA was isolated using the RNeasy mini kit (Qiagen) from *STAT1*^−/−^ SAM fibroblasts transduced with lentivirus carrying NT or RhoV-activating sgRNA, or SNB-19 cells transfected with Rho GTPase-targeting siRNAs. Input RNA (0.3 to 1 μg) was used as the template for reverse transcription using ProtoScript II First Strand cDNA synthesis (NEB) and random primer mix. RT-qPCR was performed with 5 μL of 3- to 10-fold-diluted cDNA and primers targeting Rho GTPases, ZIKV [25], or RPS11 (refer to Table 1) in a SYBR Green qPCR assay on the CFX96 Touch Real-time PCR Detection system (Bio-Rad). Primers targeting Rho GTPases were designed using pga.mgh.harvard.edu/primerbank/. qPCR conditions were as follows: initial denaturation step at 95 °C for 1 min, and then 40 cycles of 95 °C for 15 s and 60 °C for 30 s, followed by a melt curve of 0.5 °C increase from 60 to 95 °C for 10 s. RNA levels of Rho GTPases and ZIKV were determined by normalizing the target transcript C_T_ value to the C_T_ value of the endogenous housekeeping RPS11 transcript. For *STAT1*^−/−^ SAM fibroblasts transduced with lentivirus carrying RhoV-activating sgRNA, the normalized values were used to calculate the fold change in RhoV levels relative to the average of cells treated with the NT sgRNA control (C_T_ method). For SNB-19 cells treated with Rho GTPase-targeting siRNAs, the normalized values were used to calculate the fold changes in Rho GTPase and ZIKV RNA levels relative to the average of cells treated with the NT siRNA control and infected with ZIKV (C_T_ method).

## 3. Results

### 3.1. CRISPR–Cas9 Activation Screen in ZIKV-Infected Cells

**Figure 1:**
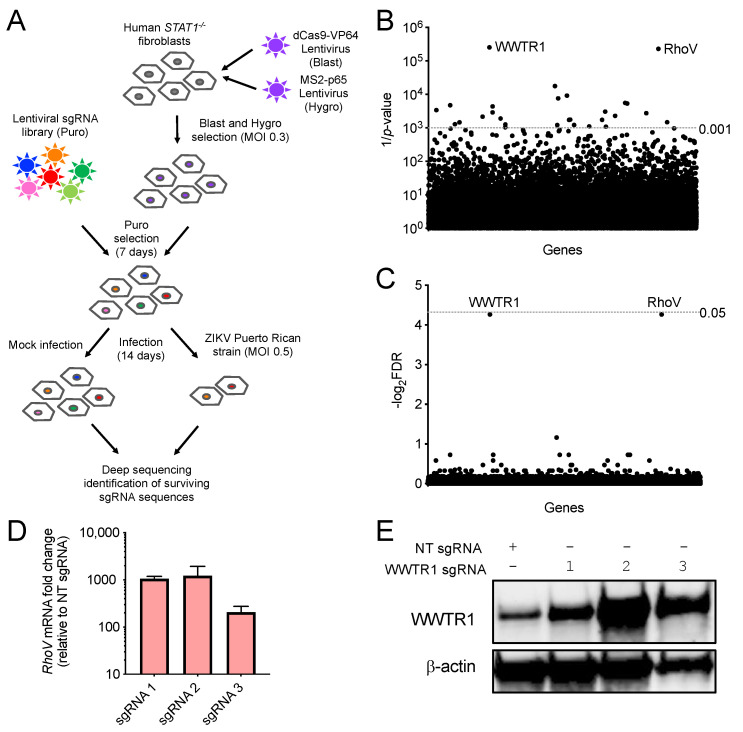
A genome-wide CRISPR activation ZIKV screen in cells defective in IFN signaling. (**A**) Schematic flow of the CRISPR activation screen setup and conditions. *STAT1*^−/−^ fibroblasts were transduced with lentiviruses carrying the SAM complex followed by the sgRNA library upregulating all known human gene isoforms. After antibiotic selection, these cells were challenged with ZIKV (strain: PRVABC59) at MOI of 0.5 PFU/cell and incubated for 14 days. Genomic DNA of mock-infected and ZIKV-infected cells was extracted, amplified, sequenced, and bioinformatically analyzed to determine potential antiviral and proviral candidate genes from the sgRNAs enriched or depleted in surviving cells, respectively. (**B**,**C**) Scatter plots showing negative selection of sgRNAs targeting the top candidate genes identified by MAGeCK VISPR, a quality control and analysis workflow for CRISPR screens, compared with other sgRNAs in the library after ZIKV infection (*p*-value < 0.001 and false discovery rate (FDR) < 0.05). (**D**) RhoV mRNA levels were measured by RT-qPCR in *STAT1*^−/−^ fibroblasts transduced with lentiviruses carrying RhoV promoter targeting sgRNAs 1, 2, and 3. mRNA fold changes relative to the RhoV mRNA levels in NT sgRNA transduced *STAT1*^−/−^ fibroblasts are shown. The data are from one independent experiment performed in triplicate. (**E**) WWTR1 protein expression in *STAT1*^−/−^ fibroblasts transduced with lentiviruses carrying WWTR1 promoter targeting sgRNAs 1, 2, and 3 as well as NT sgRNA was determined by immunoblotting using WWTR1 antibody. β-actin serves as a loading control. The data are from one experiment.

We performed a genome-wide activation screen to identify host factors important for ZIKV infection using the engineered CRISPR–Cas9 SAM complex and a sgRNA library that consists of 70,290 guides targeting every known human coding isoform [11]. This innovative approach allowed us to upregulate all gene isoforms from their endogenous promoter contexts. First, we generated in human *STAT1*^−/−^ fibroblasts stable expression of the SAM components, dCas9-VP64 and MS2-p65-HSF1, which together transcriptionally activate genes from their endogenous loci (Figure 1A). The dCas9/MS2-expressing *STAT1*^−/−^ fibroblasts were then transduced with lentiviruses carrying the entire sgRNA library, selected, and infected with ZIKV (strain: PRVABC59, MOI = 0.5 PFU/cell) or mock infected (Figure 1A). Infection conditions were optimized prior to the screen to maximize ZIKV-induced cell killing and hence selective pressure in *STAT1*^−/−^ fibroblasts. Following 14 days of infection and cell passaging, surviving cells were collected and deep sequenced to identify sgRNA sequences that were significantly selected for or against (Figure 1A). Positively and negatively selected sgRNAs in surviving ZIKV-infected cell populations target candidate genes hypothesized to activate host antiviral and proviral factors, respectively. We observed that these *STAT1*^−/−^ fibroblasts do not demonstrate high levels of cytopathic effects after infection like other cell types that are highly susceptible to ZIKV, such as A549 and HAP1 cells [9,25]. The lack of strong selective pressure imposed by ZIKV-induced cytopathic effects may explain why no sgRNAs were found to be significantly enriched in our screen (Table S1). However, we identified sgRNAs targeting two genes, Ras homolog family member V (*RhoV*) and WW domain-containing transcription regulator 1 (*WWTR1*), for which targeting sgRNAs were significantly depleted in the surviving ZIKV-infected cell populations (*p*-value < 0.001, false discovery rate (FDR) ≈ 0.05) (Figure 1B,C and Table S1).

To confirm transcriptional activation, we cloned NT, RhoV, and WWTR1 promoter targeting sgRNAs from the SAM library and introduced them into *STAT1*^−/−^ fibroblasts by lentivirus delivery. Due to the lack of a RhoV antibody, we tested the efficiency of the SAM complex to activate RhoV at its endogenous promoter by measuring RhoV mRNA levels by qPCR in the *STAT1*^−/−^ fibroblasts transduced with NT or different RhoV sgRNAs. Compared to NT sgRNA, RhoV sgRNAs 1–3 induced RhoV mRNA levels by 100- to 1000-fold when transduced in *STAT1*^−/−^ fibroblasts (Figure 1D). In addition, we confirmed that WWTR1 sgRNAs 1–3 induced WWTR1 protein levels in *STAT1*^−/−^ fibroblasts compared to NT sgRNA (Figure 1E). Taken together, these data showed that *RhoV* and *WWTR1*, proviral candidate genes for ZIKV, can be upregulated by the SAM complex in human *STAT1*^−/−^ fibroblasts.

### 3.2. Both RhoV and WWTR1 Enhance ZIKV Infection in A549 Cells

**Figure 2:**
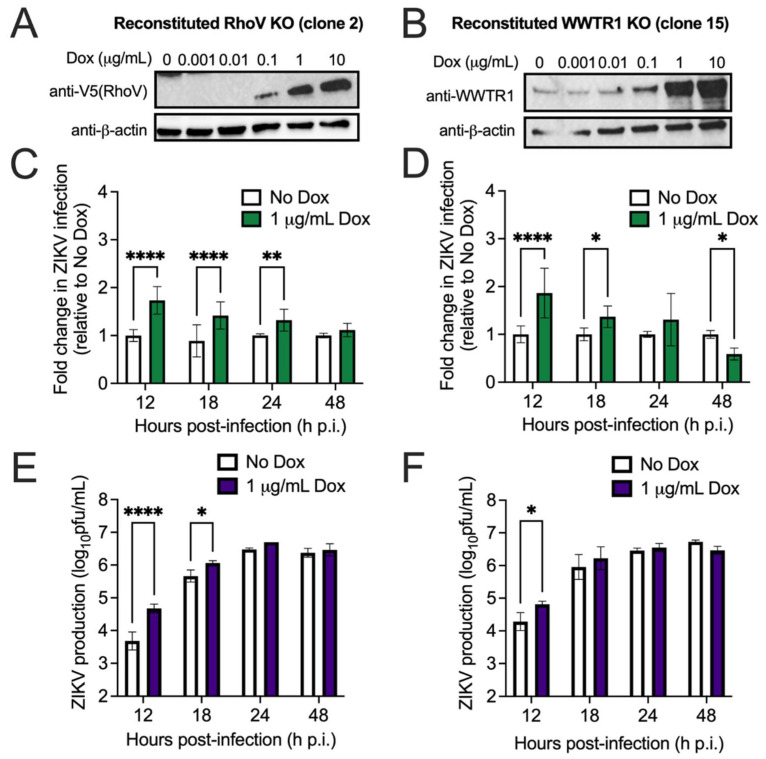
RhoV and WWTR1 enhance ZIKV infection in A549 cells. Reconstituted (**A**) RhoV or (**B**) WWTR1 A549 KO clones were treated with different amounts of Dox (0, 0.001, 0.01, 0.1, 1, and 10 µg/mL) for 24 h to induce the expression of N-terminally V5-tagged RhoV or WWTR1 through the ePiggyBac transposon system. RhoV, WWTR1, and β-actin (loading control) protein expression was determined by immunoblotting with V5, WWTR1, and β-actin antibodies. The data are representative of two independent experiments. Reconstituted (**C**) RhoV or (**D**) WWTR1 A549 KO clones were treated with or without 1 µg/mL of Dox for 24 h prior to 1 h adsorption with ZIKV (MOI = 1 PFU/cell) and harvested at 12, 18, 24, and 48 h.p.i. to quantify infection levels. Dox was added back to the media during the course of infection. ZIKV infected cells were then fixed and permeabilized to stain with the pan-flavivirus envelope antibody prior to flow cytometry analysis. Infection levels of cells treated with Dox were normalized to that of the respective untreated condition (no Dox) at each timepoint and reported as fold change in ZIKV infection. The data are combined from three independent experiments. (**E**,**F**) Supernatant of infected cells from (**C**,**D**) was collected at 12, 18, 24, and 48 h.p.i. and titered on Vero cells by plaque assay. The data are representative of three independent experiments. Asterisks indicate statistically significant differences (two-way ANOVA and Sidak’s multiple comparisons test: *, *p* < 0.05; **, *p* < 0.01; ****, *p* < 0.0001).

Next, we sought to validate the candidate host proviral factors in different cell lines. Due to its susceptibility to ZIKV infection and rapid cytopathic effects, we chose A549, a human lung adenocarcinoma cell line, to validate the proviral phenotype of RhoV and WWTR1. Many previous studies have used A549 cells as a platform to study ZIKV infection [25,31,32]. Overexpression of either RhoV or WWTR1 is expected to increase ZIKV infection or ZIKV-induced cell death. We utilized the CRISPR–Cas9 system to edit *RhoV* and *WWTR1* in bulk A549 cells and performed single cell cloning to generate KO clones. We confirmed two KO clones per gene by immunoblotting (WWTR1 only) and sequencing (both RhoV and WWTR1) (Figure S1) and set up an inducible system to express N-terminally V5-tagged RhoV and WWTR1 using a transposon-based delivery method [23,24]. These inducible cell lines were treated with different amounts of Dox to induce protein expression of RhoV (Figure 2A and Figure S2A) and WWTR1 (Figure 2B and Figure S2B). We found that 1 µg/mL Dox induces the highest achievable levels of RhoV and WWTR1 proteins, except for reconstituted RhoV KO clone 14. Therefore, we chose to induce all clones with 1 µg/mL Dox.

To investigate the effects of RhoV and WWTR1 on ZIKV infection, we treated these inducible cell lines with Dox and infected them with ZIKV. Cells were harvested and fixed at various timepoints for staining with a pan-flavivirus envelope antibody and quantification of infected cells by flow cytometry. Overexpression of RhoV (Figure 2C and Figure S2C) and WWTR1 (Figure 2D and Figure S2D) significantly enhanced ZIKV infection by up to 2.6-fold at 12 h.p.i., an effect which persisted up to 18–24 h.p.i. for RhoV (clone 2) and WWTR1 (clone 15). Overexpression of both RhoV (Figure 2E and Figure S2E) and WWTR1 (Figure 2F and Figure S2F) significantly enhanced ZIKV production by up to 17- and 4-fold at 12 h.p.i., respectively, an effect which persisted up to 18–24 h.p.i. for RhoV. Interestingly, despite the dramatic increase in virus yield in cells overexpressing RhoV at early time points that diminishes at late time points (Figure 2E and Figure S2E), RhoV does not appear essential for ZIKV infection. Additionally, we observed a significant reduction in infected cells with WWTR1 overexpression by 48 h.p.i., likely due to increased cell killing from the proviral effects of WWTR1. Based on our results in A549 cells in which RhoV has a more dramatic effect on ZIKV production (Figure 2 and Figure S2), and the recent study [17] that reported a role in ZIKV replication and neuroinflammation for the host hippo signaling pathway, in which WWTR1 is a critical player, we focused on further studying RhoV as a novel proviral factor for ZIKV infection.

### 3.3. RhoV Promotes ZIKV and DENV Infection in SNB-19 Cells

**Figure 3:**
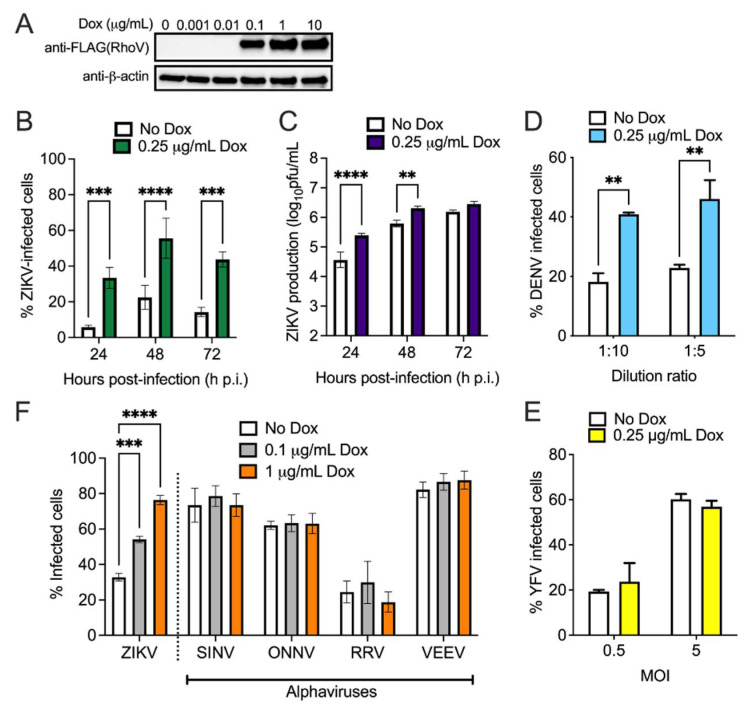
RhoV enhances ZIKV and DENV but not alphavirus infection in SNB-19 cells. (**A**) Inducible RhoV SNB-19 cells were treated with different amounts of Dox (0, 0.001, 0.01, 0.1, 1, and 10 µg/mL) for 24 h to induce the expression of N-terminally 3xFLAG-tagged RhoV through the ePiggyBac transposon system. RhoV and β-actin (loading control) protein expression determined by immunoblotting with FLAG and β-actin antibodies. The data are representative of two independent experiments. (**B**) Inducible RhoV SNB-19 cells were treated with or without 0.25 µg/mL Dox for 24 h prior to 1 h adsorption with ZIKV (MOI = 0.5 PFU/cell) and harvested at 24, 48, and 72 h.p.i. to quantify infection levels. Dox was added back to the media during the course of infection. ZIKV infected cells were then fixed and permeabilized to stain with the pan-flavivirus envelope antibody prior to flow cytometry analysis. The data are combined from two independent experiments. (**C**) Supernatant of infected cells from (**B**) was collected at the same timepoints and titered on Vero cells by plaque assay. The data are representative of two independent experiments. Inducible RhoV SNB-19 cells were treated with or without 0.25 µg/mL Dox for 24 h prior to 1 h adsorption with (**D**) DENV-GFP (1:10 or 1:5 dilution) or (**E**) YFV 17D Venus (MOI = 0.5 or 5 PFU/cell) and harvested at (**D**) 72 h.p.i. or (**E**) 48 h.p.i. to quantify infection levels. Dox was added back to the media during the course of infection. YFV- and DENV-infected cells were fixed prior to flow cytometry analysis for GFP/Venus expression. The data are representative of two independent experiments. (**F**) Inducible RhoV SNB-19 cells were treated with 0.1 and 1 µg/mL Dox 24 h prior to 1 h adsorption with ZIKV (MOI = 0.5 PFU/cell), and GFP-expressing SINV (MOI = 1 PFU/cell), ONNV (MOI = 1 PFU/cell), VEEV (MOI = 0.5 PFU/cell), and RRV (MOI = 5 PFU/cell) and harvested at 24 h.p.i. to quantify infection levels. Dox was added back to the media during the course of infection. ZIKV infected cells were then fixed and permeabilized to stain with the pan-flavivirus envelope antibody as above, while alphavirus-infected cells were fixed prior to flow cytometry analysis for GFP expression. The data are combined from two independent experiments. Asterisks indicate statistically significant differences (two-way ANOVA and (**B**–**E**) Sidak’s or (**F**) Dunnett’s multiple comparisons test: **, *p* < 0.01; ***, *p* < 0.001; ****, *p* < 0.0001).

Due to ZIKV association with microcephaly and other neurological conditions, we aimed to validate the proviral phenotype of RhoV in a cell type more relevant to the central nervous system. SNB-19 is a human glioblastoma cell line and has been used widely for studies on mechanisms of ZIKV infection and for ZIKV inhibitor screens [33,34,35]. We introduced the transposon system into SNB-19 cells to overexpress N-terminally Flag-tagged RhoV. The inducible cell line was treated with different amounts of Dox to induce protein expression (Figure 3A). Although 1 µg/mL Dox induces the highest saturating level of RhoV protein, we found that Dox amounts greater than 1 µg/mL enhances ZIKV infection non-specifically in unmodified SNB-19 cells (Figure S3A). Therefore, we chose a lower concentration of Dox (0.25 µg/mL), which has minimal non-specific effects on ZIKV infection in the inducible empty plasmid control cell line (Figure S3B).

To confirm the proviral effects of RhoV, inducible RhoV SNB-19 cells were treated with Dox and infected with ZIKV. Similar to A549 cells, infected SNB-19 cells were harvested and fixed at various timepoints for staining and quantification. Compared to untreated cells, RhoV induction significantly elevated the percentage of ZIKV infected cells at all timepoints analyzed by 2.5- to 6-fold (Figure 3B) and viral production at most timepoints analyzed by 2- to 7-fold (Figure 3C). Next, we asked if the proviral phenotype of RhoV would affect infection of other flaviviruses. We infected Dox-treated or untreated inducible RhoV SNB-19 cells with GFP-expressing DENV or Venus-expressing YFV 17D. Similar to its effects on ZIKV, RhoV overexpression significantly increased the percentage of DENV-infected cells at both virus dilutions tested (Figure 3D). Interestingly, RhoV did not affect YFV infection (Figure 3E). We then asked whether RhoV has an effect on infection of other positive-sense single-stranded RNA viruses. We treated inducible RhoV SNB-19 cells with different amounts of Dox and challenged them with both Old World (SINV, ONNV, RRV) and New World (VEEV) alphaviruses. Contrary to its proviral effects on ZIKV and DENV, RhoV overexpression did not significantly alter the percentage of alphavirus infected cells (Figure 3F). Based on these results in SNB-19 cells, the proviral effects of RhoV appear to be specific to some flaviviruses.

**Figure 4:**
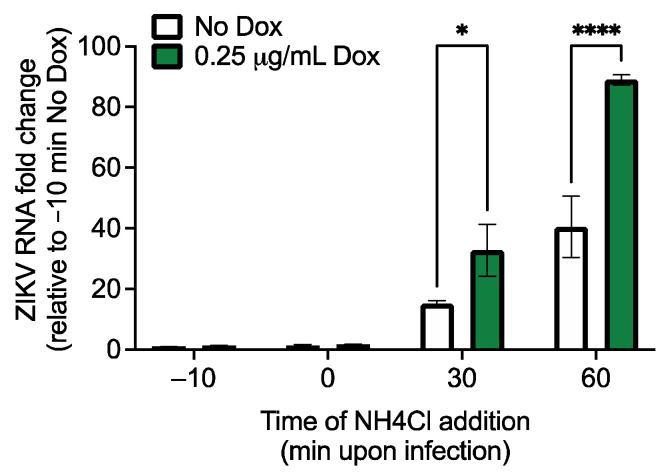
RhoV promotes endosomal entry of ZIKV. Inducible RhoV SNB-19 cells were treated with or without 0.25 µg/mL Dox for 24 h prior to infection with ZIKV (MOI = 0.5 PFU/cell). NH_4_Cl was then added to cells pre-, at, or post-virus addition (−10, 0, 30, or 60 min). After 2 h adsorption with ZIKV, the cells were washed with PBS and Dox was added back to the media during the course of infection. ZIKV-infected cells were then lysed at 24 h.p.i. for RNA extraction and reverse transcription. ZIKV RNA levels were quantified by RT-qPCR and normalized to that of untreated cells incubated with NH_4_Cl at 10 min pre-virus addition (−10 min No Dox). The data are representative of two independent experiments. Asterisks indicate statistically significant differences (two-way ANOVA and Sidak’s multiple comparisons test: *, *p* < 0.05; ****, *p* < 0.0001).

Next, we investigated which step of the ZIKV lifecycle is affected by RhoV. Since we observed a significant impact on ZIKV infection and production at early timepoints in A549 cells (Figure 2 and Figure S2) and Rho GTPases are known to modulate viral entry [36], we asked if RhoV facilitates ZIKV endosomal entry. We incubated inducible RhoV SNB-19 cells with NH_4_Cl, which prevents the reduction of pH within endosomes and therefore blocks endosomal entry of viruses, at various times pre-, at, and post-ZIKV addition. NH_4_Cl completely blocked viral entry and RNA replication when added to cells 10 min pre- or at virus addition, but increasing levels of ZIKV RNA were detected in cells over time suggesting increasing viral entry (Figure 4). Importantly, significantly higher levels of ZIKV RNA were detected in cells treated with Dox compared to untreated cells, suggesting that RhoV accelerates viral entry allowing more RNA to uncoat and replicate in the presence of NH_4_Cl.

### 3.4. The GTPase Domain Plays an Important Role in RhoV Proviral Effects

**Figure 5:**
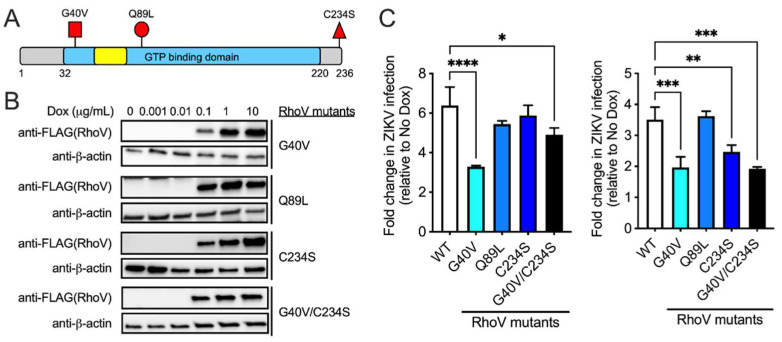
RhoV GTPase domain contributes to its proviral effects during ZIKV infection. (**A**) Schematic of RhoV protein domains and mutations introduced (red flags). The GTP binding/hydrolysis domain (blue), effector or switch I domain (yellow), and N- and C-terminal extension regions (gray) are shown. Adapted from [18]. (**B**) SNB-19 cells were treated with different amounts of Dox (0, 0.001, 0.01, 0.1, 1 and 10 µg/mL) for 24 h to induce the expression through the ePiggyBac transposon system of N-terminally 3×FLAG-tagged RhoV WT or mutants that are dominant-activated, GTPase-defective (G40V, Q89L), mislocalized (C234S), or both (G40V/C234S). Mutant RhoV and β-actin (loading control) protein expression was determined by immunoblotting with FLAG and β-actin antibodies. The data are representative of two independent experiments. (**C**) Two independent experiments are shown here (left and right). Inducible RhoV (WT or mutants) SNB-19 cells were treated with or without 0.25 µg/mL Dox for 24 h prior to 1 h adsorption with ZIKV (MOI = 0.5 PFU/cell) and harvested at 24 h.p.i. to quantify infection levels. Dox was added back to the media during the course of infection. ZIKV infected cells were then fixed and permeabilized to stain with the pan-flavivirus envelope antibody prior to flow cytometry analysis. Infection levels of RhoV WT and mutant cell lines were normalized to that of the respective untreated condition (no Dox) and reported as fold changes. Asterisks indicate statistically significant differences (one-way ANOVA and Dunnett’s multiple comparisons test: *, *p* < 0.05; **, *p* < 0.01; ***, *p* < 0.001; ****, *p* < 0.0001).

Rho GTPases bind to GTP when activated, and interact with downstream effector proteins with high affinity resulting in changes in actin cytoskeletal organization, cell cycle progression, and gene expression. This high-affinity effector-binding conformation of Rho GTPases is transient as GTP hydrolysis releases the effector proteins and suppresses downstream signaling. RhoV is an atypical Rho GTPase with N- and C-terminal extensions as well as a palmitoylated motif associated with the plasma membrane [18]. To determine which domain of RhoV is essential for its proviral effects on ZIKV infection, we generated several RhoV mutants that are mechanistically informative. We introduced two dominant-activated, GTPase-defective mutations G40V and Q89L into the core GTP binding and hydrolysis domain of RhoV (analogous to G12V and Q61L in the closely related Rho GTPase, Cdc42) (Figure 5A). Both mutations keep RhoV in the constitutively active, GTP-bound state, and we observed WT and mutants at punctate cytoplasmic locations (Figure S4), consistent with previous reports showing RhoV association with early endosomes [37]. If GTPase activity and interaction with downstream effector proteins are critical for ZIKV infection, we expect to see a further increase in RhoV proviral effects. We also introduced C234S to abrogate the palmitoylation motif [37], leading to a mislocalized RhoV (Figure 5A). If membrane targeting is critical for ZIKV infection, we expect to see a reduction in RhoV proviral effects.

Although 1–10 µg/mL Dox induces the highest achievable levels of mutant RhoV proteins (Figure 5B), we induced cells with 0.25 µg/mL Dox to minimize any non-specific proviral effects (Figure S3). Inducible WT and mutant RhoV SNB-19 cell lines were treated with Dox and infected with ZIKV in two independent experiments (Figure 5C, left and right). We found that G40V and G40V/C234S mutants increased ZIKV infection at 24 h.p.i. but to significantly lower levels compared to WT RhoV, suggesting that the proviral effects are likely dependent on an optimal level of RhoV activation similar to what was previously reported for other Rho GTPases in PDGF-induced cell invasion and transformation [38,39]. In contrast, Q89L mutant overexpression did not have a significant effect on ZIKV infection. On the other hand, abrogation of the palmitoylation motif (C234S) only affects RhoV proviral effects in one of the two experiments. Taken together, the GTPase activity plays an important role in the proviral function of RhoV during ZIKV infection. It appears that the complete GTPase cycle rather than the GTP-bound form of RhoV confers proviral activity.

### 3.5. siRNA Screen of Rho GTPases and Effector Proteins Reveals That Both RhoB and Pak1 Are Proviral Factors for ZIKV

**Figure 6:**
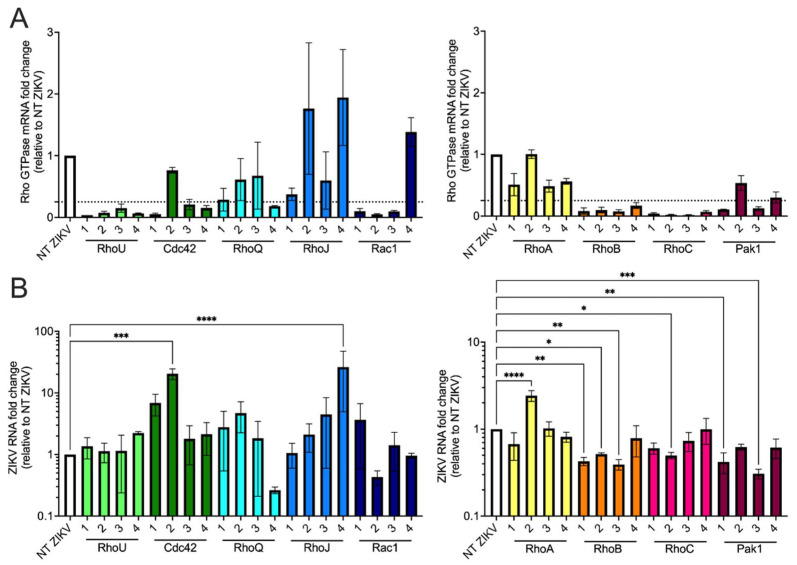
Silencing of RhoB and Pak1 negatively impacts ZIKV infection. SNB-19 cells were treated with NT, and Rho GTPase- and Pak1-targeting siRNAs for 48 h and then infected with ZIKV (MOI = 0.1 PFU/cell) for 24 h. (**A**) mRNA levels of Rho GTPases and Pak1 were measured by RT-qPCR and normalized to that of NT siRNA-treated and ZIKV-infected cells. Significant knockdown was arbitrarily set at 75% (dotted line) to uncover potential effects conferred by Rho GTPases and effector proteins. (**B**) ZIKV RNA of cells treated with Rho GTPase-targeting siRNAs in (**A**) were quantified by RT-qPCR and normalized to that of NT siRNA-treated and ZIKV-infected cells (NT ZIKV). The data are from one experiment performed in triplicate. Asterisks indicate statistically significant differences (one-way ANOVA and Dunnett’s multiple comparisons test: *, *p* < 0.05; **, *p* < 0.01; ***, *p* < 0.001; ****, *p* < 0.0001).

Previous studies showed that actin cytoskeleton reorganization, such as in filopodia and lamellipodia, monitored by small Rho GTPases, such as RhoA, Rac1, and Cdc42, is critical for supporting flavivirus entry and release in host cells [40,41,42,43]. Furthermore, it was known that RhoV activates protein kinase Pak1 [44], and Pak1 major functions include actin cytoskeleton reorganization [45]. These findings suggest that Pak1 and RhoV may be involved in the same pathway to regulate actin cytoskeleton. Therefore, we asked whether other related Rho GTPases and RhoV effector proteins such as Pak1 are also able to enhance ZIKV infection. SNB-19 cells were treated with NT or Rho GTPase-targeting siRNAs (Figure 6) and challenged with ZIKV. Total RNA was harvested from the cells for RT-qPCR to quantify Rho GTPase gene knockdown and ZIKV RNA levels. Rho GTPase mRNA levels were comparable between mock- and ZIKV-infected NT siRNA-treated cells (data not shown). All gene knockdown results were normalized to the NT siRNA-treated ZIKV-infected (NT ZIKV) SNB-19 cells. We chose the arbitrary cutoff of 75% knockdown (Figure 6A, dotted line) as indicative of sufficient protein depletion that might uncover phenotypes caused by reduction of Rho GTPase or related protein. We found that at least two out of four siRNAs efficiently introduce 75% gene knockdown for *RhoU*, *Cdc42*, *Rac1*, *RhoB*, *RhoC*, and *Pak1* (Figure 6A). While most of these siRNAs with efficient knockdown did not lead to significant changes in viral RNA levels, *RhoB* (siRNAs 1–3) and *Pak1* (siRNAs 1 and 3) knockdown significantly decreased ZIKV RNA levels compared to NT ZIKV cells in a way that correlated with the extent of gene knockdown (Figure 6B). In summary, these data support that RhoB and Pak1, in addition to RhoV, are potential proviral factors for ZIKV.

## 4. Discussion

In this study, we identified RhoV and WWTR1 as candidate host proviral factors for ZIKV infection by performing a genome-wide CRISPR activation screen, which has the advantage of upregulating all gene isoforms from their endogenous promoter contexts [11]. We found that RhoV overexpression enhances ZIKV infection and production at multiple time points in *RhoV* KO A549 cell clones. Since WWTR1, a critical player in the host hippo signaling pathway, has been reported to play a role in ZIKV replication and neuroinflammation [17], we focused on validating RhoV as a novel proviral factor for ZIKV. RhoV overexpression in SNB-19 cells significantly increases ZIKV and DENV infection, although it does not affect alphavirus infection. This pro-flavivirus activity of RhoV appears to act at the step of endosomal entry. Further investigation identified the GTPase domain of RhoV to be critical for its proviral phenotype, while siRNA-mediated knockdown of RhoB, another Rho GTPase, and Pak1, the direct effector of RhoV, leads to reduced ZIKV infection. This enhancement of virus infection by Pak1 suggests Pak1 might participate in the same pathway that RhoV uses to support the ZIKV lifecycle in host cells. Taken together, these data indicate that RhoV, an atypical Rho GTPase, has previously uncharacterized proviral effects that are likely mediated through its GTPase activity and its downstream effector protein, Pak1.

Small Rho GTPases are known to be involved in supporting flavivirus entry and release in host cells [40,41,42,43]. Consistent with previous studies, we found that RhoV facilitates ZIKV endosomal entry and its proviral effects are specific for flaviviruses. Further studies are needed to define the exact contribution of RhoV signaling to flavivirus entry, which is likely dependent on the entry route and cell type involved. Nevertheless, RhoV provides an attractive therapeutic target as it acts early in the viral life cycle. Interestingly, RhoV promotes DENV infection at both virus dilutions tested but has no effects on YFV infection, suggesting that cytoskeleton reorganization by RhoV might affect related viruses differently. Not surprisingly, ZIKV is phylogenetically more related to DENV than YFV within the flavivirus genus [46]. We also investigated the impact of RhoV on alphavirus infection as alphaviruses, like flaviviruses, enter host cells through receptor-mediated endocytosis as well as fusion employing acidic endosomes [47,48,49]. Therefore, alphaviruses might utilize similar Rho GTPases to enter host cells. Interestingly, our data show that RhoV does not enhance the infection of alphaviruses (SINV, ONNV, RRV, and VEEV), suggesting that RhoV’s proviral effects are unique to flaviviruses. Rac1, another Rho GTPase, has been shown to act at a late stage of VEEV infection prior to viral budding through regulating actin cytoskeleton, and that knockdown of Rac1 in HeLa cells reduces viral titer by 10- to more than 30-fold [50].

Since RhoV is an atypical Rho GTPase that is targeted to plasma membrane through palmitoylation [18], we tested dominant-activated, GTPase-defective (G40V and Q89L), or mislocalized (C234S) mutants for their ability to increase ZIKV infection. We found that the G40V mutation significantly reduces the proviral phenotype of RhoV, which is contrary to what we expected for a constitutively active RhoV. These data suggest that the complete GTPase cycle rather than the GTP-bound form of RhoV is required for proviral activity as shown previously for other Rho GTPases such as Cdc42, RhoA, and Rac1 in cell invasion and transformation [38,39]. While both G40V and Q89L are found in the GTP binding and hydrolysis domain, Q89L is located in the homologous switch II domain of Cdc42 and could affect RhoV interactions with multiple downstream effector proteins [37,51]. This might explain why this mutation preserves the ability of RhoV to facilitate ZIKV infection. For other flaviviruses, it was shown that the expression of the dominant-negative GTPase mutants of Rac1 and Cdc42 in HMEC-1 cells specifically inhibits the formation of filopodia induced by DENV-2 leading to a reduction in viral infection and titer [41,42]. Further studies are needed to elucidate how GTPase activity of RhoV and interactions with downstream effector proteins contribute to its proviral effects during ZIKV infection.

RhoV activates serine/threonine kinase Pak1 and induces actin cytoskeleton reorganization [45] as well as ubiquitin-dependent degradation of Pak1 [44]. Therefore, we investigated whether other Rho GTPases and effector protein of RhoV, Pak1, carry similar functions as RhoV during ZIKV infection in SNB-19 cells. We found that knockdown of RhoB or Pak1 significantly decreases ZIKV RNA levels, suggesting that RhoB also acts as a potential proviral factor for ZIKV, and RhoV might enhance viral entry through activating Pak1. These data suggest that there are other Rho GTPases with redundant proviral effects as RhoV, and is in line with our results in RhoV KO A549 cells where RhoV does not appear essential for ZIKV infection despite the dramatic increase in virus yield upon RhoV overexpression at early time points. RhoB is not only a component of the human cytomegalovirus (HCMV) assembly complex but is also required for productive viral infection. RhoB knockdown in HCMV-infected cells significantly reduces viral titer [52,53]. In addition, RhoB overexpression enhances infection of viruses pseudotyped with either Ebola or vesicular stomatitis viral glycoprotein [54]. Our findings align with many previous studies reporting that small Rho GTPases play diverse roles in many stages of the viral lifecycle for both DNA and RNA viruses [36].

In conclusion, we have uncovered RhoV as a novel and specific proviral factor for ZIKV through a CRISPR transcriptional activation screen and follow-up studies using cell lines with inducible expression of RhoV and mechanistically informative mutants. This proviral activity is dependent on the GTPase activity of RhoV and its effector kinase Pak1. Our findings advance our understanding of host–flavivirus interactions and provide an attractive therapeutic target for treating ZIKV infection. Since RhoV is an atypical Rho GTPase, therapeutic targeting will likely pose minimal side effects as the closely related Rho GTPases have redundant cellular functions. Finally, this study demonstrates the power of CRISPR activation in an IFN signaling-defective cellular context to uncover and validate novel host–pathogen interactions.

## Figures and Tables

**Table 1:** qPCR primers.

Gene Name	Forward (F) and Reverse (R) Primer Sequences
*RhoV*	F: 5′-CCTCATCGTCAGCTACACCTG-3′ R: 5′-GAACGAAGTCGGTCAAAATCCT-3′
*RhoU*	F: 5′-GCTACCCCACCGAGTACATC-3′ R: 5′-GGCTCACGACACTGAAGCA-3′
*Cdc42*	F: 5′-CCATCGGAATATGTACCGACTG-3′ R: 5′-CTCAGCGGTCGTAATCTGTCA-3′
*RhoQ*	F: 5′-CCACCGTCTTCGACCACTAC-3′ R: 5′-AGGCTGGATTTACCACCGAGA-3′
*RhoJ*	F: 5′-AGGGGCAACGACGAGAAGA-3′ R: 5′-TTGGCGTAGCTCATCAGCAG-3′
*Rac1*	F: 5′-ATGTCCGTGCAAAGTGGTATC-3′ R: 5′-CTCGGATCGCTTCGTCAAACA-3′
*RhoA*	F: 5′-GGAAAGCAGGTAGAGTTGGCT-3′ R: 5′-GGCTGTCGATGGAAAAACACAT-3′
*RhoB*	F: 5′-CTGCTGATCGTGTTCAGTAAGG-3′ R: 5′-TCAATGTCGGCCACATAGTTC-3′
*RhoC*	F: 5′-GGAGGTCTACGTCCCTACTGT-3′ R: 5′-CGCAGTCGATCATAGTCTTCC-3′
*Pak1*	F: 5′-CAGCCCCTCCGATGAGAAATA-3′ R: 5′-CAAAACCGACATGAATTGTGTGT-3′
*ZIKV*	F: 5′-TTGTGGAAGGTATGTCAGGTG-3′ R: 5′-ATCTTACCTCCGCCATGTTG-3′
*RPS11*	F: 5′-GCCGAGACTATCTGCACTAC-3′ R: 5′-ATGTCCAGCCTCAGAACTTC-3′

## Supplementary Materials

The following are available online at https://www.mdpi.com/article/10.3390/v13112113/s1, Figure S1: Validation of *RhoV* and *WWTR1* CRISPR KO A549 clones, Figure S2: RhoV and WWTR1 enhance ZIKV infection in A549 cells, Figure S3: Dox titration to determine the amount with minimal non-specific effects, Figure S4: Constitutively active mutants of RhoV found in punctate cytoplasmic locations similar as WT, Table S1: ZIKV SAM screen results.
