# Supplementary material for: A CRISPR Activation Screen Identifies an Atypical Rho GTPase That Enhances Zika Viral Entry
Source: Viruses. 2021 Oct 20;13(11):2113. doi: 10.3390/v13112113 (PMC8623001; doi:10.3390/v13112113)
Supplement: Supplementary file 1 [file viruses-13-02113-s001.zip › Supplemental Figures_FINAL.pdf]

A

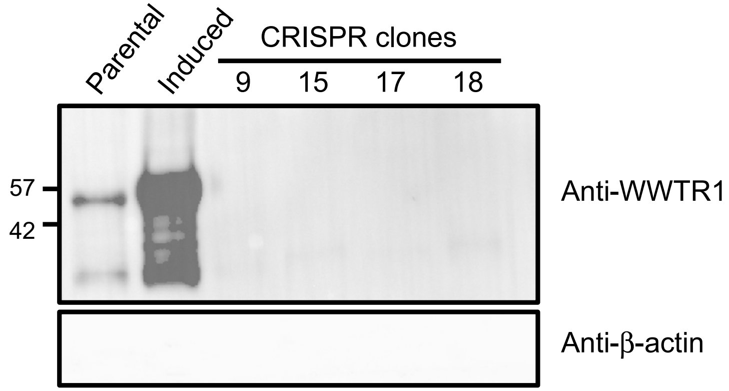

B

RhoV KO clone 2

```
ACACCTGCAATGGGTACCCCGCGCTACCGGCCCACTGCGCTGGACACCTTCTCTGG WT
ACACCTGCAATGGGTACCCCGCGCT----------------GGACACCTTCTCTGG 91.62%
```

RhoV KO clone 14

```
ACACCTGCAATGGGTACCCCGCGCTACCGGCCCACTGCGCTGGACACCTTCTCTGG WT
ACACCTGCAATGGGTACCCCG-----TACCGGCCCACTGCGCTGGACACCTTCTCTGG 98.34%
```

WWTR1 KO clone 15

```
GCAAGTGATCCACGTCA-CGCAGGACCTAGACACAGACCTCGAAGCCCTCTTCAACTCTGTCATGAATCCGAAGCCTAGCTCGTGGCGGAAGAAGATCCTGCCGGAGTCTTTCTTTAAGGAGCCTGATTCGGGCTCGCACTCGCGCCAGTCCAGCACCGACTCGTCGGGCGGCCACCCGG WT
GCAAGTGATACACGTCA-CGCAGGACCTAGACACAGACCTCGAAGCCCTCTTCAACTCTGTCATGAATCCGAAGCCTAGCTCGTGGCGGAAGAAGATCCTGCCGGAGTCTTTCTTTAAGGAGCCTGATTCGGGCTCGCACTCGCGCCAGTCCAGCACC----------------ACCCGG 35.16%
GCAAGTGATCCACGTCA-CGCAGGACCTAGACACAGACCTCGAAGCCCTCTTCAACTCTGTCATGAATCCGAAGCCTAGCTCGTGGCGGAAGAAGATCCTGCCGGAGTCTTTCTTTAAGGAGCCTGATTCGGGCTCGCACTCGCGCCAGTCCAGCACCGACT-GTCGGGCGGCCACCCGG 33.08%
GCAAGTGATCCACGTCATCGCAGGACCTAGACACAGACCTCGAAGCCCTCTTCAACTCTGTCATGAATCCGAAGCCTAGCTCGTGGCGGAAGAAGATCCTGCCGGAGTCTTTCTTTAAGGAGCCTGATTCGGGCTCGCACTCGCGCCAGTCCAGCACCGACTCGTCGGGCGGCCACCCGG 22.0%
GCAAGTGATCTACGTCA-CGCAGGACCTAGACACAGACCTCGAAGCCCTCTTCAACTCTGTCATGAATCCGAAGCCTAGCTCGTGGCGGAAGAAGATCCTGCCGGAGTCTTTCTTTAAGGAGCCTGATTCGGGCTCGCACTCGCGCCAGTCCAGCACCGACTCGTCGGGCGGCCACCCGG 2.32%
GAAAGTGATCCACGTCA-CGCAGGACCTAGACACAGACCTCGAAGCCCTCTTCAACTCTGTCATGAATCCGAAGCCTAGCTCGTGGCGGAAGAAGATCCTGCCGGAGTCTTTCTTTAAGGAGCCTGATTCGGGCTCGCACTCGCGCCAGTCCAGCACCGACTCGTCGGGCGGCCACCCGG 2.32%
```

WWTR1 KO clone 17

```
GCAAGTGATCCACGTCA-CGCAGGACCTAGACACAGACCTCGAAGCCCTCTTCAACTCTGTCATGAATCCGAAGCCTAGCTCGTGGCGGAAGAAGATCCTGCCGGAGTCTTTCTTTAAGGAGCCTGATTCGGGCTCGCACTCGCGCCAGTCCAGCACCGACTCGTCGGGCGGCCACCCG WT
GCAAGTGATCCACGTC---------------------------------------------------------------------------------------------------------------------------------------------------GTCGGGCGGCCACCCG 87.16%
GCAAGTGATCCACGTCATCGCAGGACCTAGACACAGACCTCGAAGCCCTCTTCAACTCTGTCATGAATCCGAAGCCTAGCTCGTGGCGGAAGAAGATCCTGCCGGAGTCTTTCTTTAAGGAGCCTGATTCGGGCTCGCACTCGCGCCAGTCCAGCACCGACT-GTCGGGCGGCCACCCG 5.2%
GCAAGTGATCCACGTCATCGCAGTACCTAGACACAGACCTCGAAGCCCTCTTCAACTCTGTCATGAATCCGAAGCCTAGCTCGTGGCGGAAGAAGATCCTGCCGGAGTCTTTCTTTAAGGAGCCTGATTCGGGCTCGCACTCGCGCCAGTCCAGCACCGACT-GTCGGGCGGCCACCCG 3.36%
```

Figure S1. Validation of *RhoV* and *WWTR1* CRISPR KO A549 clones. (**A**) WWTR1 protein levels in parental A549 cells (Parental), A549 cells with inducible expression of V5-tagged WWTR1 (Induced), and CRISPR clones 9, 15, 17, and 18 were detected by immunoblotting using an anti-WWTR1 antibody. The protein levels of WWTR1 in clones 9, 15, 17, and 18 are undetectable compared to that in parental A549 cells. β-actin was used as a loading control. Endogenous WWTR1 protein: 44kDa. (**B**) CRISPR-targeting region in the genomic sequence of *RhoV* is shown for clones 2 and 14. CRISPR-targeting region in the genomic sequence of *WWTR1* is shown for clones 15 and 17, of which WWTR1 proteins are undetectable in (**A**). The top sequence for each clone shows the WT sequence of *RhoV* or *WWTR1*. The alignment shown is in the same reading frame as the WT RhoV or WWTR1 protein. A red dash represents a deletion whereas a red nucleotide represents a substitution/insertion when compared to the WT RhoV or WWTR1 sequence. *RhoV* KO clones 2 and 14 have a premature stop codon in all of their alleles. *WWTR1* KO clone 15 has a premature stop codon in its 3 dominant alleles. *WWTR1* KO clone 17 has a premature stop codon in all of its alleles.

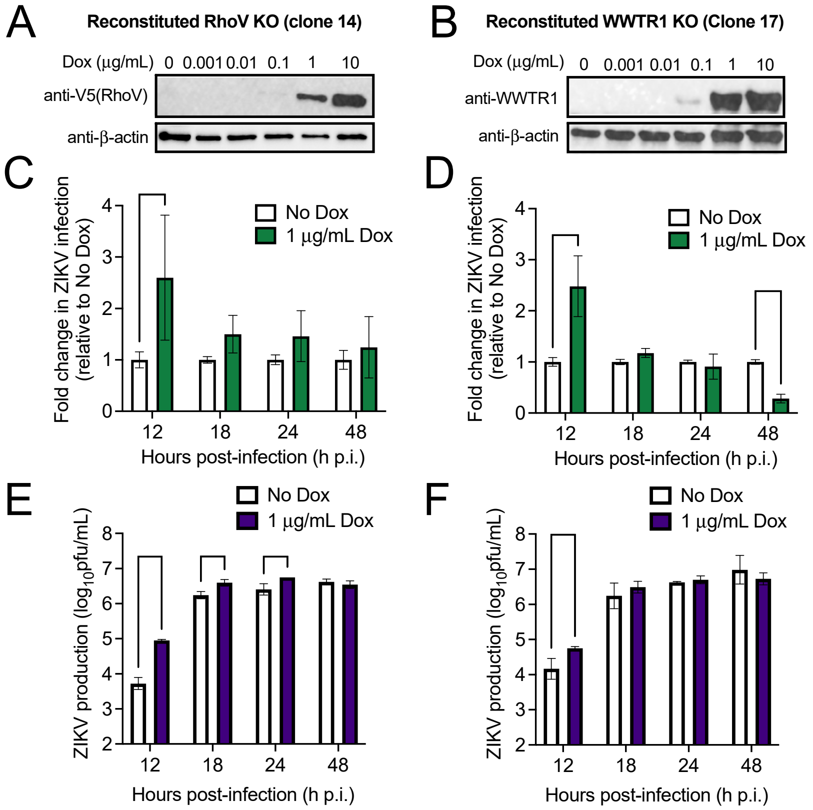

Figure S2. RhoV and WWTR1 enhance ZIKV infection in A549 cells. Reconstituted (**A**) RhoV or (**B**) WWTR1 A549 KO clones were treated with different amounts of Dox (0, 0.001, 0.01, 0.1, 1, and 10 µg/mL) for 24 h to induce the expression of N-terminally V5-tagged RhoV or WWTR1 through the ePiggyBac transposon system. RhoV, WWTR1, and β-actin (loading control) protein expression was determined by immunoblotting with V5, WWTR1, and β-actin antibodies. The data is representative of two independent experiments. Reconstituted (**C**) RhoV or (**D**) WWTR1 A549 KO clones were treated with or without 1 µg/mL of Dox for 24 h prior to 1 h adsorption with ZIKV (MOI = 1 PFU/cell) and harvested at 12, 18, 24, and 48 h.p.i. to quantify infection levels. Dox was added back to the media during the course of infection. ZIKV infected cells were then fixed and permeabilized to stain with the pan-flavivirus envelope antibody prior to flow cytometry analysis. Infection levels of cells treated with Dox were normalized to that of the respective untreated condition (no Dox) at each timepoint and reported as fold change in ZIKV infection. The data is combined from three independent experiments. (**E-F**) Supernatant of infected cells from (**C-D**) was collected at 12, 18, 24, and 48 h.p.i. and titered on Vero cells by plaque assay. The data is a representative of three independent experiments. Asterisks indicate statistically significant differences (Two-way ANOVA and Sidak's multiple comparisons test: *, $p<0.05$; **, $p<0.01$; ****, $p<0.0001$).

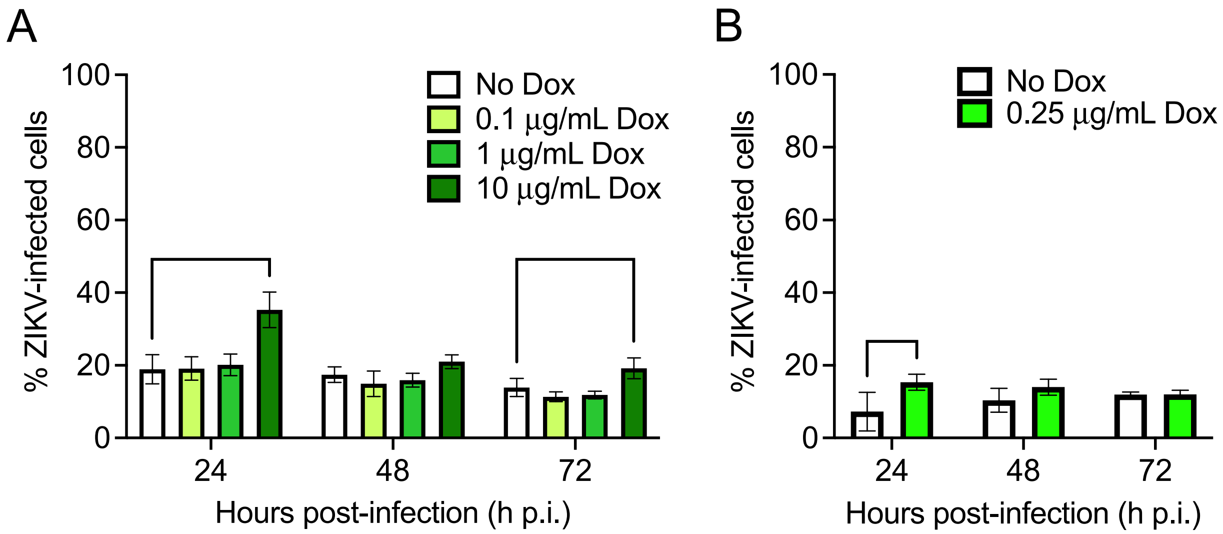

Figure S3. Dox titration to determine the amount with minimal non-specific effects. (**A**) SNB-19 cells were treated with different amounts of Dox (0, 0.1, 1, 10 µg/mL) 24 h prior to 1 h adsorption with ZIKV (MOI = 0.5 PFU/cell) and harvested at 24, 48, and 72 h.p.i. for flow cytometry analysis to quantify infection levels. Respective amounts of Dox were added back to the media during the course of infection. The data is combined from two independent experiments. (**B**) SNB-19 cells with inducible expression of 3xFLAG ePB construct (control) were treated with or without 0.25 µg/mL of Dox for 24 h prior to 1 h adsorption with ZIKV (MOI = 0.5 PFU/cell) and harvested at 24, 48, and 72 h.p.i. to quantify infection levels. Dox was added back to the media during the course of infection. The data is representative of two independent experiments. ZIKV infected cells in (**A**) and (**B**) were fixed and permeabilized to stain with the pan-flavivirus envelope antibody prior to flow cytometry analysis. Asterisks indicate statistically significant differences (Two-way ANOVA and (**A**) Dunnett's or (**B**) Sidak's multiple comparison test: *, $p<0.05$; **, $p<0.01$; ****, $p<0.0001$).

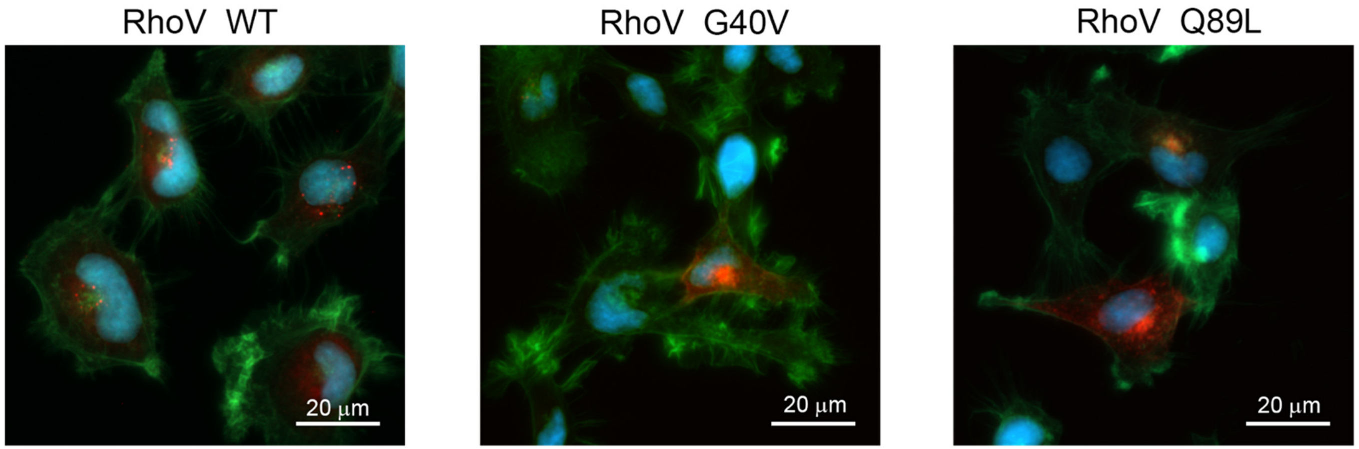

Figure S4. Constitutively active mutants of RhoV found in punctate cytoplasmic locations similar as WT. SNB-19 cells overexpressing 3xFLAG-tagged RhoV WT and mutants (G40V and Q89L) were treated with 1 μg/mL Dox for 24 h to induce RhoV expression. The cells were fixed, permeabilized, and incubated with primary anti-FLAG antibody followed by secondary Alexa Fluor 594-conjugated antibody, and phalloidin-iFluor 488, which stains for actin filaments, and DAPI, which stains for nuclei. Red: RhoV; green: actin filaments; blue: nuclei.
